# Rheumatism and Gout

**Published:** 1904-12-10

**Authors:** 


### RHEUMATISM AND GOUT.

**Rheumatoid Arthritis.**—Ewart[1] reports some cases treated by "interrupted circulation." The limb is elevated for a few moments and stroked downwards to empty the veins, a band of padding is wrapped round it, and a rubber loop drawn tightly about it as a tourniquet to compress all the vessels till the limb is blanched and numb in from 30 to 120 seconds. The tourniquet is then suddenly loosed and a flush appears. After a few seconds the manœuvre is repeated for, perhaps, half-a-dozen times. It appears that much of the effusion and swelling around the joints disappears under the daily application of this treatment, probably from the forcible arterial flushing alternating with the suctional tissue drainage. Relapsing acute articular rheumatism, gonorrhœal rheumatism, and the early stages of rheumatoid arthritis are benefited by the addition of this method to the ordinary treatment. The numbness caused by such a tourniquet has been found to relieve the pain of dressing burns, and the alternating compression has been used to give force to the circulation in commencing gangrene after ligature of a wounded femoral artery. C. E. Skinner, of New Haven[2] recommends the combined use of a very full meat diet, rest in bed for at least 10 hours a day, ferrous iodide and strychnine in pill form with cod-liver oil in emaciated cases, hot air, central galvanisation and vibratory stimulation two or three times a week, static electricity, and in quiescent cases passive movements daily. Great improvement, and in some cases a practical cure, had been thus obtained. C. J. Macalister[3] draws attention to the various modes in which the disease may commence. Sometimes there is at first a fever like that of true rheumatism but unaffected by salicylates. It may, however, yield to quinine in large doses or guaiacol, and there may be rhinitis, bronchiectasis, gastric or uterine trouble which is the probable source of the infection. After the fever has subsided progressive arthritis sets in. In one instance the disease followed an attack of scarlatina, and in others it has been connected with myxœdema or Raynaud's disease, or exophthalmic goître. Indeed, asthma, migraine and cramps are sometimes seen in addition to the ordinary symptoms, and Raynaud's disease is often seen in persons whose near relatives suffer from rheumatoid arthritis. Discussion, too, has taken place as to whether some cases at least of the latter disease are a special form of tuberculosis of the joints, and the rarefaction of the ends of the bones in skiagrams has been mentioned in favour of this view. The types described as chronic rheumatism are thought by Edsall and Lavenson to be those most connected with tuberculosis. R. Llewellyn Jones[4] considers it a cerebro-spinal toxæmia, and gives cases showing its connection with Raynaud's disease and with tetany, as well as 15 instances of its combination with Grave's disease. Merrins[5] distinguishes osteo-arthritis as a secondary affection following (*a*) toxic diseases such as tubercle, tabes, syphilis, rheumatism, rheumatoid arthritis, and gout, through the lingering irritation of toxins of these diseases after an imperfect recovery. (*b*) Impaired nutrition in, for example, disease of the pituitary body, or thymus or in osteo-malacia. (*c*) Injury, such as blows, strains, or hard work in elderly people where the powers of repair are feeble. S. H. Perry[6] insists on the recognition of acute as well as chronic forms. The former is often mistaken for rheumatic fever, but is followed by permanent joint changes. Liberal diet, hot baths, such as the Aix douche with massage, iodide of iron with arsenic, and a dose of salicylates at night to relieve the pains are specially useful.

**Hypertrophic Osteoarthropathy.**—An exhaustive and critical account with bibliography based on 100 definite cases is given by W. H. Wynn.[7] He notices the symmetrical enlargement of the hands and feet, clubbing of fingers, thickening of diaphyses, effusions in joints and curvature of the nails, with similar changes in the bones and joints of the arms and legs, and sometimes elsewhere, as well as muscular wasting and skin affections. Most of the cases were preceded by lung troubles, but others by heart or liver disease, typhus, syphilis, and intestinal disease. One factor seems common to nearly all, viz. auto-intoxication from the alimentary canal. Simple clubbing of the fingers, is, he thinks, only an early stage of the same condition. *Periodic hydrarthrosis* is another rare affection, in which pain and swelling occur in one or more joints at fixed intervals. Howard Marsh[8] has discussed 48 recorded cases, where the average duration of an attack was four days, and the average recurrence once a fortnight, though in one case there was a daily attack. The effusion is never purulent, but there may be synovial thickening. Sometimes the swelling and pain have disappeared under quinine and arsenic, but whether the affection is a neurosis, or what is its cause, is entirely unknown.

**Spondylose Rhizomélique.** — Gordon[9] mentions two forms of this disease, (*a*) ankylosis of the cervical spine with meningo-myelitis, (*b*) ankylosis of the spine and large joints without any affection of the cord. The former type occurs after injury in those who have an hereditary disposition towards it, and is confined to the upper part of the cord and spine. The type of Strumpell and Marie on the other hand extends more widely and shows the changes of osteo-arthritis. Gordon describes three cases showing that the symptoms of both may be combined, and believes that the symptoms may arise in the course of various diseases, such as tabes and syringomyelia. Fiorentini[10] remarks that spinal ankylosis may be due to chronic meningitis, syphilis, caries of the spine, gonorrhœal and ordinary rheumatism, acromegaly, syringomyelia, and finally to this spondylosis. He reports a case which was due to rheumatism, but he regards the condition as produced by trophic disturbances set up by various poisons.

**Gout.**—Von Noorden[11] at the Oxford meeting said that an excess of nitrogen and of phosphoric acid is eliminated during attacks, showing the presence of an endogenous toxin. The symptoms result from the retention of uric acid in the system, and he thinks that the normal individual has the power of forming certain organic substances which dissolve out uric acid. A. E. Garrod holds that it is definitely proved that there is an excess of uric acid in the blood during an attack. This excess depends, he thinks, on retention, and in support of that view he mentioned that interstitial nephritis was present in the kidneys of all the post-mortems of gouty patients during a year at St. Mary's hospital. Ringrose Gore refused to give so much importance to uric acid, for there is, in some cases, little or no excess, and hardly ever the amount seen in leukæmia. Walker Hall looks on uric acid only as a symptom and not as a cause of the disease. On the whole he thinks there is some alteration in the construction and affinities of the endogenous purins. Both forms of purins are in excess in the tissues, whereas normally about half these bodies, whether endogenous or exogenous, should be excreted unchanged, and the other half as urea or intermediate bodies. In his book on the purin bodies he has pointed out how to estimate the changes. Chalmers Watson[12] insists that there is probably an infective element in gout. Ebstein and Garrod both hold that uric acid is the primary irritant, but the former thinks that the acid in a liquid form first causes necrosis of tissues, and that afterwards a crystalline deposit is formed around the dead matter, whereas Garrod thinks the deposition of the salt is the cause of the inflammatory change. Watson cannot regard these explanations as sufficient. He finds that gouty deposits contain other material besides crystals, which is possibly more important; that necrotic areas along the course of the blood-vessels can be detected, and that the erosion of cartilage is due to the action of masses of small round cells. The bone-marrow is profoundly altered and also the leucocytes. In fact, the histology shows a close resemblance to changes found in chronic infective diseases. Sir Dyce Duckworth[13] draws attention to the importance of metastatic attacks of gout, though they do not appear so common as a century ago. Indeed, the same may be said of gout itself. He gives an instance of violent gastric pains continuing for weeks until an articular attack appeared, when the stomach pains vanished. Then again there may be severe cerebral apoplectiform attacks, or orchitis, or throat trouble, or cystitis, or violent dyspepsia. The head symptoms may simulate uræmic attacks, but they all disappear when a joint is involved. If a hot mustard bath or mustard plasters round the feet are applied the visceral pains will often be at once replaced by an attack in the joints. The patient should then be carefully dieted and treated with colchicum and potash. When recovering quinine and iodide of potash together are often most helpful.

1 Lancet, Aug. 13. 2 Med. Rec., June 25. 3 Lancet, July 23. 4 Edinburgh Med. Jour., vol. xv., p. 425. 5 Med. News, p. 152. 6 Birmingham Med. Rev., March. 7 Ibid. 8 Lancet, June 11. 9 Edinburgh Med. Jour., July. 10 Med. Rec., Aug. 13. 11 Lancet, Aug. 20. 12 Brit. Med. Jour., July 16. 13 Clin. Jour., No. 603.

